# 25,26,27,28-Tetra­propoxycalix[4]arene-5,17-dicarbonitrile

**DOI:** 10.1107/S1600536810002242

**Published:** 2010-01-23

**Authors:** Jan Budka, Václav Eigner, Roman Holakovský, Petr Kovaříček, Tereza Loužilová

**Affiliations:** a Department of Organic Chemistry, Institute of Chemical Technology, Prague, Technická 5, 166 28, Prague 6, Czech Republic; b Department of Solid State Chemistry, Institute of Chemical Technology, Prague, Technická 5, 166 28, Prague 6, Czech Republic

## Abstract

In the title compound, C_42_H_46_N_2_O_4_, both crystallographically independent mol­ecules display a 1,3-alternate conformation. Their crystal packing is stabilized by non-classical C—H⋯N hydrogen bonds. The dihedral angles between the planes of the aromatic rings and the mean plane through the methyl­ene C atoms bridging the aromatic rings are 78.10 (13), 80.74 (14), 81.89 (12) and 79.05 (14)° for the first mol­ecule, and 71.65 (11), 76.60 (13), 77.97 (14) and 74.76 (13)° for the second mol­ecule. Both mol­ecules have three C atoms of one prop­oxy chain disordered over two set of sites; the site-occupancy factors are 0.7/0.3 and 0.6/0.4, respectively.

## Related literature

For calix[4]arene derivatives and their uses as supra­molecular building blocks, see: Gutsche (2008 ▶); Rao & Dey (2004 ▶). For applications of the title compound, see: Pinkhassik *et al.* (1997 ▶; 1998 ▶). For details of the synthesis, see: Sýkora *et al.* (2005 ▶); Casnati *et al.* (1996 ▶). For the weighting scheme, see: Prince (1982 ▶); Watkin (1994 ▶).
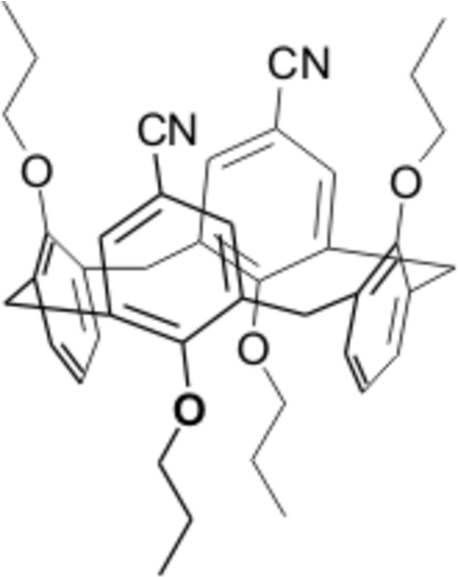


## Experimental

### 

#### Crystal data

C_42_H_46_N_2_O_4_
*M* *_r_* = 642.84
Monoclinic, 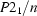

*a* = 19.398 (4) Å
*b* = 10.6491 (12) Å
*c* = 34.391 (2) Å
β = 93.987 (10)°
*V* = 7086.9 (16) Å^3^
*Z* = 8
Cu *K*α radiation
μ = 0.61 mm^−1^
*T* = 150 K
0.40 × 0.33 × 0.12 mm

#### Data collection

Oxford Diffraction XCALIBUR diffractometer
Absorption correction: analytical (de Meulenaer & Tompa, 1965 ▶) *T*_min_ = 0.83, *T*_max_ = 0.93
44656 measured reflections
14658 independent reflections
7906 reflections with *I* > 2σ(*I*)
*R*_int_ = 0.103

#### Refinement

*R*[*F* ^2^ > 2σ(*F* ^2^)] = 0.083
*wR*(*F* ^2^) = 0.063
*S* = 1.10
7906 reflections
920 parameters
18 restraints
H-atom parameters constrained
Δρ_max_ = 0.38 e Å^−3^
Δρ_min_ = −0.41 e Å^−3^

### 

Data collection: *CrysAlis CCD* (Oxford Diffraction, 2002 ▶); cell refinement: *CrysAlis RED* (Oxford Diffraction, 2002 ▶); data reduction: *CrysAlis RED*; program(s) used to solve structure: *SIR92* (Altomare *et al.*, 1994 ▶); program(s) used to refine structure: *CRYSTALS* (Betteridge *et al.*, 2003 ▶); molecular graphics: *CAMERON* (Watkin *et al.*, 1996 ▶); software used to prepare material for publication: *CRYSTALS*.

## Supplementary Material

Crystal structure: contains datablocks global, I. DOI: 10.1107/S1600536810002242/om2307sup1.cif

Structure factors: contains datablocks I. DOI: 10.1107/S1600536810002242/om2307Isup2.hkl

Additional supplementary materials:  crystallographic information; 3D view; checkCIF report

## Figures and Tables

**Table 1:** Hydrogen-bond geometry (Å, °)

*D*—H⋯*A*	*D*—H	H⋯*A*	*D*⋯*A*	*D*—H⋯*A*
C10—H101⋯N30^i^	0.95	2.55	3.392 (8)	147

Symmetry code: (i) 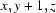.

**Fig. 1.**
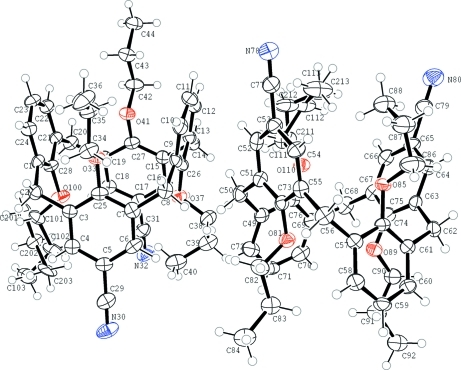
The title compound with displacement ellipsoids drawn at the 50% probability level. H atoms are shown as spheres of arbitrary radius.

## supplementary crystallographic information

### Comment

Calix[4]arene is one of the most important molecular scaffolds used for preparation of structurally well defined ligands (Gutsche, 2008; Rao & Dey, 2004). Calix[4]arene in 1,3 alternate conformation bearing two nitrile groups was prepared from appropriate bromo derivative (Sýkora *et al.*, 2005) by heating with CuCN in *N*-methylpyrrolidone (Casnati *et al.*, 1996). Such molecules can be used as a starting material for preparation of ligands for recognition of neutral molecules (Pinkhassik *et al.*, 1998) or neutral molecules and silver cation (Pinkhassik *et al.*, 1997).

The title compound adopts 1,3-alternate conformation (Fig. 1) with all phenolic rings pitched away from the calix cavity, as defined by the angles which the aromatic rings make with the plane of the four bridging methylenes 78.10 (13), 80.74 (14), 81.89 (12) and 79.05 (14)° for the first and 71.65 (11), 76.60 (13), 77.97 (14) and 74.76 (13)° for the second molecule. Two opposite rings make interplanar angle of 20.03 (15) and 20.21 (18)° for the first molecule and 30.38 (18) and 28.64 (16)° for the second.

In the crystal structure there are non-classical intermolecular hydrogen bonds (Table 1).

### Experimental

A mixture of 5,17-dibromo-25,26,27,28-tetrapropoxycalix[4]arene (1 g, 1.33 mmol) and CuCN (0.48 g, 5.32 mmol) in *N*-methylpyrrolidone (20 ml) was refluxed for 5 h and cooled to 373 K. Then reaction was quenched by careful addition of a solution of FeCl_3_ (4.32 g, 26.64 mmol) in HCl (2 *M*, 30 ml) and resulting mixture was refluxed for 1 h. Reaction mixture was filtrated and filtration cake was washed by methanol. The solvent was removed under reduced pressure and the residue was purified by chromatography (silica gel, dichloromethane) to give the title compound as a white solid (38% yield). Crystals of the title compound suitable for X-ray diffraction were obtained by recrystallization from pentan-1-ol.

### Refinement

In the absence of significant anomalous scattering, Friedel pairs were merged.

The H atoms were positioned geometrically. The H atoms were initially refined with soft restraints on the bond lengths and angles to regularize their geometry (C—H in the range 0.93–0.98 = 0.82 Å) and *U*_iso_(H) (in the range 1.2–1.5 times *U*_eq_ of the parent atom), after which the positions were refined with riding constraints.

### Crystal data

**Table:**

C_42_H_46_N_2_O_4_	*F*(000) = 2752
*M**_r_* = 642.84	*D*_x_ = 1.205 Mg m^−^^3^
Monoclinic, *P*2_1_/*n*	Cu *K*α radiation, λ = 1.54184 Å
*a* = 19.398 (4) Å	Cell parameters from 3818 reflections
*b* = 10.6491 (12) Å	θ = 4–78°
*c* = 34.391 (2) Å	µ = 0.61 mm^−^^1^
β = 93.987 (10)°	*T* = 150 K
*V* = 7086.9 (16) Å^3^	Block, colorless
*Z* = 8	0.40 × 0.33 × 0.12 mm

### Data collection

**Table:**

Oxford Diffraction XCALIBUR diffractometer	14658 independent reflections
graphite	7906 reflections with *I* > 2σ(*I*)
Detector resolution: 8.1917 pixels mm^-1^	*R*_int_ = 0.103
φ & ω scans	θ_max_ = 78.0°, θ_min_ = 4.4°
Absorption correction: analytical (de Meulenaer & Tompa, 1965)	*h* = −24→24
*T*_min_ = 0.83, *T*_max_ = 0.93	*k* = −13→10
44656 measured reflections	*l* = −43→43

### Refinement

**Table:**

Refinement on *F*	Primary atom site location: structure-invariant direct methods
Least-squares matrix: full	Hydrogen site location: inferred from neighbouring sites
*R*[*F*^2^ > 2σ(*F*^2^)] = 0.083	H-atom parameters constrained
*wR*(*F*^2^) = 0.063	Method, part 1, Chebychev polynomial, (Watkin, 1994, *P*rince, 1982) [*w*eight] = 1.0/[A_0_*T_0_(x) + A_1_*T_1_(x) ··· + A_n-1_]*T_n-1_(x)] where A_i_ are the Chebychev coefficients listed belo*w* and x = *F* /*F*max Method = Robust Weighting (*P*rince, 1982) W = [*w*eight] * [1-(delta*F*/6*sigma*F*)^2^]^2^ A_i_ are: 20.7 2.42 17.0 5.57
*S* = 1.10	(Δ/σ)_max_ = 0.025
7906 reflections	Δρ_max_ = 0.38 e Å^−^^3^
920 parameters	Δρ_min_ = −0.41 e Å^−^^3^
18 restraints	

### Special details

**Table:**

Experimental. The crystal was placed in the cold stream of an Oxford Cryosystems open-flow nitrogen cryostat (Cosier & Glazer, 1986) with a nominal stability of 0.1 K.Cosier, J. & Glazer, A.*M*., 1986. J. Appl. Cryst. 105 107.

### Fractional atomic coordinates and isotropic or equivalent isotropic displacement parameters (Å^2^)

**Table:**

	*x*	*y*	*z*	*U*_iso_*/*U*_eq_	Occ. (<1)
C1	−0.16556 (18)	0.4376 (3)	0.23298 (9)	0.0275	
C2	−0.18252 (17)	0.3717 (3)	0.27001 (9)	0.0311	
C3	−0.12130 (17)	0.3503 (3)	0.29917 (8)	0.0271	
C4	−0.10537 (18)	0.2313 (3)	0.31249 (9)	0.0304	
C5	−0.04849 (19)	0.2113 (3)	0.33919 (9)	0.0311	
C6	−0.00659 (19)	0.3116 (3)	0.35112 (9)	0.0321	
C7	−0.02107 (18)	0.4323 (3)	0.33784 (8)	0.0309	
C8	0.02424 (18)	0.5405 (3)	0.35143 (9)	0.0317	
C9	0.05738 (17)	0.6155 (3)	0.32052 (9)	0.0290	
C10	0.0464 (2)	0.7449 (4)	0.31806 (10)	0.0384	
C11	0.0760 (2)	0.8175 (4)	0.28992 (11)	0.0431	
C12	0.1154 (2)	0.7594 (4)	0.26308 (11)	0.0408	
C13	0.12687 (18)	0.6316 (3)	0.26431 (9)	0.0304	
C14	0.16828 (19)	0.5669 (3)	0.23429 (10)	0.0342	
C15	0.13041 (18)	0.4641 (3)	0.21166 (9)	0.0291	
C16	0.15761 (19)	0.3438 (3)	0.21164 (9)	0.0329	
C17	0.12149 (19)	0.2453 (3)	0.19366 (9)	0.0329	
C18	0.05767 (18)	0.2655 (3)	0.17417 (9)	0.0315	
C19	0.02885 (18)	0.3857 (3)	0.17266 (8)	0.0289	
C20	−0.04104 (18)	0.4070 (3)	0.15165 (8)	0.0321	
C21	−0.09681 (17)	0.4562 (3)	0.17646 (8)	0.0274	
C22	−0.12971 (19)	0.5685 (3)	0.16751 (9)	0.0331	
C23	−0.18030 (19)	0.6160 (3)	0.18957 (10)	0.0345	
C24	−0.19669 (19)	0.5492 (3)	0.22246 (9)	0.0324	
C25	−0.07966 (18)	0.4501 (3)	0.31274 (8)	0.0286	
C26	0.09994 (18)	0.5601 (3)	0.29420 (9)	0.0270	
C27	0.06721 (18)	0.4841 (3)	0.19084 (9)	0.0298	
C28	−0.11674 (17)	0.3890 (3)	0.20906 (9)	0.0269	
C29	−0.0336 (2)	0.0874 (4)	0.35388 (10)	0.0401	
N30	−0.0225 (2)	−0.0119 (3)	0.36587 (10)	0.0520	
C31	0.1504 (2)	0.1185 (4)	0.19617 (10)	0.0398	
N32	0.17173 (19)	0.0195 (4)	0.19923 (11)	0.0533	
O33	−0.09751 (12)	0.5706 (2)	0.30097 (6)	0.0307	
C34	−0.1406 (2)	0.6342 (4)	0.32702 (10)	0.0389	
C35	−0.1527 (2)	0.7663 (4)	0.31376 (11)	0.0447	
C36	−0.1926 (3)	0.8391 (4)	0.34246 (13)	0.0589	
O37	0.11401 (12)	0.4343 (2)	0.29594 (6)	0.0318	
C38	0.1672 (2)	0.3982 (4)	0.32532 (10)	0.0408	
C39	0.1975 (2)	0.2771 (4)	0.31373 (12)	0.0498	
C40	0.1457 (3)	0.1729 (4)	0.30550 (13)	0.0569	
O41	0.03734 (12)	0.6013 (2)	0.18968 (6)	0.0317	
C42	0.0610 (2)	0.6836 (3)	0.15999 (10)	0.0393	
C43	0.0217 (2)	0.8039 (3)	0.16186 (10)	0.0381	
C44	0.0428 (2)	0.8993 (4)	0.13194 (10)	0.0423	
C49	0.17093 (19)	0.5917 (3)	0.43020 (9)	0.0317	
C50	0.10659 (19)	0.6705 (3)	0.43161 (9)	0.0349	
C51	0.10631 (18)	0.7754 (3)	0.46104 (9)	0.0307	
C52	0.08556 (18)	0.8947 (4)	0.44904 (10)	0.0349	
C53	0.08259 (18)	0.9916 (3)	0.47558 (10)	0.0325	
C54	0.09891 (19)	0.9688 (3)	0.51519 (10)	0.0331	
C55	0.12074 (18)	0.8517 (3)	0.52829 (9)	0.0306	
C56	0.13606 (19)	0.8299 (4)	0.57130 (9)	0.0355	
C57	0.20346 (18)	0.7647 (3)	0.58309 (8)	0.0283	
C58	0.20567 (19)	0.6579 (3)	0.60630 (9)	0.0330	
C59	0.2675 (2)	0.5972 (3)	0.61645 (9)	0.0346	
C60	0.32872 (19)	0.6413 (3)	0.60260 (9)	0.0319	
C61	0.32819 (17)	0.7484 (3)	0.57941 (8)	0.0270	
C62	0.39482 (17)	0.7946 (3)	0.56404 (9)	0.0298	
C63	0.39364 (17)	0.8188 (3)	0.52035 (9)	0.0284	
C64	0.41060 (18)	0.9367 (3)	0.50711 (9)	0.0320	
C65	0.41053 (18)	0.9598 (3)	0.46702 (9)	0.0324	
C66	0.39656 (18)	0.8650 (4)	0.44069 (9)	0.0343	
C67	0.37943 (17)	0.7453 (3)	0.45318 (9)	0.0310	
C68	0.3638 (2)	0.6409 (4)	0.42359 (10)	0.0391	
C69	0.2944 (2)	0.5771 (3)	0.42600 (9)	0.0327	
C70	0.2899 (2)	0.4486 (4)	0.42981 (10)	0.0413	
C71	0.2271 (2)	0.3895 (4)	0.43268 (10)	0.0437	
C72	0.1679 (2)	0.4625 (3)	0.43377 (9)	0.0393	
C73	0.12332 (17)	0.7547 (3)	0.50084 (9)	0.0276	
C74	0.26602 (17)	0.8110 (3)	0.57113 (8)	0.0249	
C75	0.37822 (18)	0.7248 (3)	0.49324 (9)	0.0306	
C76	0.23460 (19)	0.6481 (3)	0.42400 (8)	0.0286	
C77	0.0631 (2)	1.1155 (4)	0.46194 (11)	0.0386	
N78	0.04860 (18)	1.2140 (3)	0.45042 (10)	0.0490	
C79	0.4238 (2)	1.0858 (4)	0.45415 (10)	0.0397	
N80	0.43288 (19)	1.1876 (4)	0.44481 (10)	0.0534	
O81	0.14712 (12)	0.6391 (2)	0.51289 (6)	0.0324	
C82	0.0958 (2)	0.5522 (3)	0.52443 (11)	0.0370	
C83	0.1314 (2)	0.4333 (4)	0.53708 (11)	0.0415	
C84	0.0813 (2)	0.3380 (4)	0.55222 (12)	0.0476	
O85	0.26478 (12)	0.9211 (2)	0.54991 (6)	0.0280	
C86	0.2820 (3)	1.0317 (4)	0.57184 (12)	0.0621	
C87	0.2569 (3)	1.1425 (4)	0.55306 (12)	0.0538	
C88	0.2576 (2)	1.1503 (4)	0.50982 (12)	0.0531	
O89	0.35794 (12)	0.6090 (2)	0.50624 (6)	0.0325	
C90	0.4122 (2)	0.5221 (4)	0.51573 (11)	0.0388	
C91	0.3820 (2)	0.4065 (4)	0.53278 (12)	0.0440	
C92	0.4371 (2)	0.3138 (4)	0.54757 (14)	0.0558	
O100	−0.08424 (14)	0.2768 (2)	0.21819 (7)	0.0406	
C101	−0.1158 (5)	0.1686 (8)	0.2079 (2)	0.0378	0.7000
C102	−0.0723 (4)	0.0606 (6)	0.2238 (2)	0.0466	0.7000
C103	−0.1108 (4)	−0.0648 (6)	0.2199 (2)	0.0561	0.7000
C201	−0.1361 (12)	0.166 (2)	0.1935 (6)	0.0459	0.3000
C202	−0.0973 (7)	0.0466 (11)	0.1968 (4)	0.0362	0.3000
C203	−0.0836 (9)	−0.0023 (15)	0.2385 (5)	0.0506	0.3000
O110	0.23755 (13)	0.7757 (2)	0.41760 (6)	0.0315	
C111	0.2177 (8)	0.813 (2)	0.3791 (6)	0.0365	0.6000
C112	0.2513 (6)	0.9416 (12)	0.3704 (3)	0.0536	0.6000
C113	0.2363 (9)	1.0425 (14)	0.4005 (3)	0.0650	0.6000
C211	0.2384 (13)	0.805 (4)	0.3753 (9)	0.0373	0.4000
C212	0.2127 (9)	0.9321 (18)	0.3702 (5)	0.0555	0.4000
C213	0.2559 (14)	1.022 (2)	0.3899 (5)	0.0709	0.4000
H81	−0.0036	0.5967	0.3650	0.0408*	
H82	0.0602	0.5085	0.3688	0.0408*	
H341	−0.1187	0.6341	0.3526	0.0554*	
H342	−0.1839	0.5927	0.3272	0.0554*	
H352	−0.1092	0.8059	0.3119	0.0589*	
H351	−0.1773	0.7664	0.2889	0.0589*	
H363	−0.1996	0.9233	0.3340	0.0756*	
H361	−0.1679	0.8386	0.3673	0.0756*	
H362	−0.2360	0.7991	0.3443	0.0756*	
H22	−0.2146	0.4233	0.2823	0.0406*	
H21	−0.2033	0.2930	0.2635	0.0406*	
H241	−0.2301	0.5827	0.2385	0.0429*	
H231	−0.2031	0.6925	0.1825	0.0438*	
H221	−0.1176	0.6135	0.1451	0.0413*	
H201	−0.0352	0.4661	0.1314	0.0363*	
H202	−0.0565	0.3294	0.1406	0.0363*	
H421	0.1089	0.6999	0.1654	0.0511*	
H422	0.0538	0.6465	0.1349	0.0511*	
H432	0.0292	0.8388	0.1872	0.0490*	
H431	−0.0259	0.7853	0.1569	0.0490*	
H443	0.0164	0.9740	0.1337	0.0561*	
H442	0.0904	0.9183	0.1369	0.0561*	
H441	0.0352	0.8648	0.1065	0.0561*	
H142	0.2082	0.5312	0.2475	0.0458*	
H141	0.1819	0.6282	0.2163	0.0458*	
H121	0.1354	0.8083	0.2437	0.0501*	
H111	0.0692	0.9059	0.2892	0.0591*	
H101	0.0178	0.7839	0.3359	0.0467*	
H161	0.2019	0.3283	0.2243	0.0451*	
H181	0.0340	0.1971	0.1615	0.0396*	
H41	−0.1331	0.1621	0.3036	0.0401*	
H61	0.0324	0.2973	0.3688	0.0413*	
H381	0.1473	0.3883	0.3496	0.0502*	
H382	0.2018	0.4614	0.3276	0.0502*	
H391	0.2294	0.2501	0.3343	0.0678*	
H392	0.2213	0.2913	0.2909	0.0678*	
H403	0.1689	0.0988	0.2982	0.0689*	
H402	0.1218	0.1568	0.3281	0.0689*	
H401	0.1137	0.1980	0.2848	0.0689*	
H721	0.1246	0.4238	0.4370	0.0534*	
H711	0.2245	0.3006	0.4341	0.0560*	
H701	0.3309	0.3997	0.4301	0.0533*	
H681	0.3991	0.5792	0.4273	0.0468*	
H682	0.3651	0.6763	0.3983	0.0468*	
H901	0.4445	0.5586	0.5345	0.0515*	
H902	0.4350	0.5012	0.4930	0.0515*	
H912	0.3549	0.4302	0.5535	0.0580*	
H911	0.3535	0.3665	0.5129	0.0580*	
H921	0.4161	0.2415	0.5579	0.0754*	
H922	0.4657	0.3533	0.5675	0.0754*	
H923	0.4643	0.2895	0.5269	0.0754*	
H641	0.4210	1.0028	0.5251	0.0421*	
H661	0.3985	0.8809	0.4136	0.0468*	
H622	0.4071	0.8709	0.5771	0.0367*	
H621	0.4293	0.7332	0.5703	0.0367*	
H862	0.2619	1.0248	0.5962	0.0896*	
H861	0.3308	1.0365	0.5761	0.0896*	
H871	0.2852	1.2093	0.5633	0.0684*	
H872	0.2108	1.1554	0.5598	0.0684*	
H882	0.2405	1.2296	0.5009	0.0667*	
H883	0.3034	1.1394	0.5022	0.0667*	
H881	0.2289	1.0855	0.4987	0.0667*	
H581	0.1635	0.6261	0.6148	0.0415*	
H591	0.2680	0.5256	0.6329	0.0437*	
H601	0.3710	0.5986	0.6089	0.0410*	
H562	0.1356	0.9092	0.5840	0.0431*	
H561	0.1000	0.7789	0.5800	0.0431*	
H822	0.0723	0.5860	0.5454	0.0484*	
H821	0.0636	0.5358	0.5030	0.0484*	
H832	0.1658	0.4522	0.5573	0.0553*	
H831	0.1526	0.3982	0.5155	0.0553*	
H842	0.1051	0.2633	0.5601	0.0616*	
H843	0.0601	0.3731	0.5738	0.0616*	
H841	0.0469	0.3190	0.5321	0.0616*	
H501	0.0703	0.6150	0.4372	0.0507*	
H502	0.0970	0.7062	0.4065	0.0507*	
H521	0.0729	0.9107	0.4223	0.0425*	
H541	0.0938	1.0348	0.5334	0.0421*	
H1011	−0.1593	0.1652	0.2190	0.0678*	0.7000
H1012	−0.1225	0.1604	0.1804	0.0678*	0.7000
H1022	−0.0613	0.0758	0.2508	0.0602*	0.7000
H1021	−0.0310	0.0585	0.2105	0.0602*	0.7000
H1031	−0.0833	−0.1325	0.2301	0.0671*	0.7000
H1033	−0.1522	−0.0604	0.2330	0.0671*	0.7000
H1032	−0.1219	−0.0777	0.1928	0.0671*	0.7000
H2011	−0.1814	0.1640	0.2023	0.0699*	0.3000
H2012	−0.1383	0.1906	0.1668	0.0699*	0.3000
H2022	−0.1261	−0.0083	0.1811	0.0589*	0.3000
H2021	−0.0529	0.0501	0.1868	0.0589*	0.3000
H2033	−0.0637	−0.0835	0.2370	0.0599*	0.3000
H2032	−0.1274	−0.0087	0.2491	0.0599*	0.3000
H2031	−0.0542	0.0498	0.2547	0.0599*	0.3000
H1111	0.1688	0.8206	0.3755	0.0458*	0.6000
H1112	0.2334	0.7542	0.3610	0.0458*	0.6000
H1122	0.3000	0.9306	0.3724	0.0800*	0.6000
H1121	0.2370	0.9714	0.3451	0.0800*	0.6000
H1133	0.2590	1.1191	0.3953	0.0631*	0.6000
H1132	0.2508	1.0146	0.4261	0.0631*	0.6000
H1131	0.1877	1.0554	0.3988	0.0631*	0.6000
H2112	0.2829	0.7886	0.3665	0.0458*	0.4000
H2111	0.2055	0.7504	0.3624	0.0458*	0.4000
H2121	0.1655	0.9420	0.3754	0.0897*	0.4000
H2122	0.2182	0.9483	0.3435	0.0897*	0.4000
H2132	0.2398	1.1036	0.3829	0.0699*	0.4000
H2131	0.2506	1.0083	0.4169	0.0699*	0.4000
H2133	0.3033	1.0146	0.3850	0.0699*	0.4000

### Atomic displacement parameters (Å^2^)

**Table:**

	*U*^11^	*U*^22^	*U*^33^	*U*^12^	*U*^13^	*U*^23^
C1	0.0296 (18)	0.031 (2)	0.0216 (14)	0.0004 (15)	−0.0024 (12)	−0.0020 (13)
C2	0.0264 (19)	0.037 (2)	0.0306 (16)	−0.0001 (16)	0.0047 (13)	0.0016 (15)
C3	0.0315 (19)	0.030 (2)	0.0204 (14)	0.0001 (15)	0.0075 (13)	0.0012 (13)
C4	0.034 (2)	0.035 (2)	0.0234 (15)	0.0012 (16)	0.0076 (13)	−0.0026 (14)
C5	0.041 (2)	0.029 (2)	0.0242 (15)	0.0053 (16)	0.0093 (14)	0.0000 (14)
C6	0.033 (2)	0.041 (2)	0.0230 (15)	0.0141 (16)	0.0059 (13)	0.0029 (15)
C7	0.037 (2)	0.039 (2)	0.0172 (14)	−0.0026 (16)	0.0088 (13)	−0.0047 (14)
C8	0.034 (2)	0.038 (2)	0.0237 (15)	0.0014 (16)	0.0048 (13)	−0.0069 (14)
C9	0.0288 (19)	0.0247 (19)	0.0327 (17)	−0.0021 (15)	−0.0032 (13)	−0.0081 (14)
C10	0.039 (2)	0.036 (2)	0.0401 (19)	0.0013 (17)	−0.0023 (16)	−0.0119 (16)
C11	0.051 (3)	0.025 (2)	0.052 (2)	−0.0012 (18)	−0.0068 (19)	−0.0071 (17)
C12	0.045 (2)	0.036 (2)	0.041 (2)	−0.0083 (18)	−0.0032 (17)	0.0004 (17)
C13	0.034 (2)	0.028 (2)	0.0290 (16)	−0.0029 (15)	−0.0012 (14)	−0.0029 (14)
C14	0.031 (2)	0.037 (2)	0.0351 (18)	−0.0022 (16)	0.0083 (14)	0.0027 (15)
C15	0.0303 (19)	0.036 (2)	0.0216 (14)	−0.0021 (15)	0.0092 (13)	0.0027 (13)
C16	0.031 (2)	0.041 (2)	0.0280 (16)	0.0037 (16)	0.0072 (14)	0.0057 (15)
C17	0.037 (2)	0.038 (2)	0.0251 (16)	0.0053 (17)	0.0092 (14)	0.0040 (15)
C18	0.038 (2)	0.034 (2)	0.0232 (15)	0.0019 (16)	0.0087 (14)	−0.0037 (14)
C19	0.0335 (19)	0.037 (2)	0.0169 (14)	0.0001 (16)	0.0072 (13)	0.0011 (13)
C20	0.039 (2)	0.040 (2)	0.0180 (14)	0.0022 (16)	0.0030 (13)	0.0010 (14)
C21	0.0304 (19)	0.031 (2)	0.0201 (14)	−0.0009 (15)	−0.0011 (13)	0.0016 (13)
C22	0.037 (2)	0.037 (2)	0.0242 (15)	−0.0016 (16)	−0.0014 (14)	0.0038 (14)
C23	0.040 (2)	0.028 (2)	0.0344 (17)	0.0117 (16)	−0.0011 (15)	0.0011 (15)
C24	0.038 (2)	0.030 (2)	0.0294 (16)	0.0043 (16)	0.0038 (14)	−0.0026 (14)
C25	0.040 (2)	0.030 (2)	0.0173 (14)	0.0038 (16)	0.0109 (13)	0.0021 (13)
C26	0.0305 (19)	0.0220 (19)	0.0281 (16)	−0.0004 (14)	−0.0011 (13)	−0.0024 (13)
C27	0.036 (2)	0.034 (2)	0.0212 (15)	0.0044 (16)	0.0116 (14)	0.0058 (13)
C28	0.0305 (19)	0.0250 (19)	0.0252 (15)	0.0087 (15)	0.0019 (13)	0.0028 (13)
C29	0.051 (3)	0.044 (3)	0.0253 (16)	0.0104 (19)	0.0039 (15)	−0.0024 (16)
N30	0.072 (3)	0.041 (2)	0.0420 (18)	0.0242 (19)	−0.0011 (17)	−0.0006 (16)
C31	0.041 (2)	0.046 (3)	0.0321 (18)	0.003 (2)	0.0026 (15)	0.0038 (16)
N32	0.049 (2)	0.045 (2)	0.065 (2)	0.0191 (19)	−0.0049 (17)	0.0016 (18)
O33	0.0414 (15)	0.0279 (13)	0.0232 (10)	0.0030 (11)	0.0047 (10)	0.0024 (9)
C34	0.044 (2)	0.043 (2)	0.0306 (17)	0.0147 (18)	0.0074 (15)	0.0033 (16)
C35	0.056 (3)	0.038 (2)	0.0394 (19)	0.0112 (19)	−0.0041 (18)	−0.0023 (17)
C36	0.066 (3)	0.052 (3)	0.057 (3)	0.030 (2)	−0.006 (2)	−0.007 (2)
O37	0.0391 (15)	0.0288 (14)	0.0275 (11)	0.0032 (11)	0.0026 (10)	0.0007 (9)
C38	0.044 (2)	0.043 (2)	0.0352 (18)	0.0048 (18)	−0.0037 (16)	0.0031 (16)
C39	0.054 (3)	0.051 (3)	0.044 (2)	0.013 (2)	0.0024 (19)	0.0054 (19)
C40	0.070 (3)	0.045 (3)	0.056 (2)	0.014 (2)	0.005 (2)	0.006 (2)
O41	0.0383 (14)	0.0306 (14)	0.0276 (11)	0.0004 (11)	0.0118 (10)	0.0037 (10)
C42	0.052 (2)	0.037 (2)	0.0300 (17)	0.0028 (18)	0.0151 (16)	0.0094 (15)
C43	0.049 (2)	0.030 (2)	0.0355 (18)	−0.0023 (17)	0.0093 (16)	0.0004 (15)
C44	0.060 (3)	0.028 (2)	0.0386 (19)	−0.0007 (18)	0.0012 (18)	0.0003 (16)
C49	0.041 (2)	0.030 (2)	0.0222 (15)	0.0008 (16)	−0.0051 (14)	−0.0034 (14)
C50	0.038 (2)	0.039 (2)	0.0269 (16)	−0.0055 (17)	−0.0033 (14)	−0.0018 (15)
C51	0.0286 (19)	0.033 (2)	0.0297 (16)	0.0015 (15)	−0.0012 (13)	−0.0006 (14)
C52	0.0254 (19)	0.047 (2)	0.0320 (17)	−0.0038 (17)	−0.0007 (14)	−0.0012 (16)
C53	0.031 (2)	0.032 (2)	0.0342 (18)	0.0021 (16)	0.0016 (14)	0.0015 (15)
C54	0.036 (2)	0.033 (2)	0.0303 (17)	0.0088 (16)	0.0031 (14)	−0.0010 (15)
C55	0.0283 (19)	0.037 (2)	0.0278 (16)	−0.0020 (16)	0.0068 (13)	−0.0034 (14)
C56	0.039 (2)	0.041 (2)	0.0279 (16)	0.0060 (17)	0.0082 (15)	−0.0026 (15)
C57	0.034 (2)	0.032 (2)	0.0191 (14)	−0.0037 (15)	0.0070 (13)	−0.0006 (13)
C58	0.038 (2)	0.040 (2)	0.0222 (15)	−0.0029 (17)	0.0081 (14)	0.0013 (14)
C59	0.047 (2)	0.030 (2)	0.0265 (16)	−0.0071 (17)	0.0035 (15)	0.0082 (14)
C60	0.037 (2)	0.034 (2)	0.0249 (15)	−0.0007 (16)	0.0022 (14)	0.0054 (14)
C61	0.0341 (19)	0.0292 (19)	0.0180 (14)	−0.0039 (15)	0.0037 (12)	−0.0012 (13)
C62	0.0295 (19)	0.037 (2)	0.0229 (15)	−0.0089 (15)	0.0027 (13)	0.0015 (14)
C63	0.0231 (18)	0.037 (2)	0.0249 (15)	−0.0024 (15)	0.0002 (13)	0.0026 (14)
C64	0.031 (2)	0.036 (2)	0.0293 (16)	−0.0015 (16)	0.0040 (14)	0.0024 (15)
C65	0.0289 (19)	0.037 (2)	0.0321 (17)	0.0031 (16)	0.0067 (14)	0.0099 (15)
C66	0.029 (2)	0.049 (2)	0.0255 (16)	0.0022 (17)	0.0062 (14)	0.0069 (16)
C67	0.0268 (19)	0.043 (2)	0.0244 (15)	0.0051 (16)	0.0085 (13)	0.0006 (15)
C68	0.041 (2)	0.049 (2)	0.0275 (16)	0.0065 (19)	0.0042 (15)	−0.0047 (16)
C69	0.046 (2)	0.028 (2)	0.0242 (15)	0.0038 (16)	0.0013 (14)	−0.0048 (14)
C70	0.055 (3)	0.038 (2)	0.0303 (17)	0.015 (2)	−0.0027 (16)	−0.0054 (16)
C71	0.076 (3)	0.023 (2)	0.0314 (18)	0.001 (2)	−0.0038 (18)	−0.0026 (15)
C72	0.062 (3)	0.031 (2)	0.0247 (16)	−0.0077 (19)	−0.0020 (16)	−0.0042 (14)
C73	0.0228 (18)	0.030 (2)	0.0295 (16)	0.0001 (14)	0.0015 (13)	0.0010 (14)
C74	0.0316 (19)	0.0210 (18)	0.0221 (14)	0.0026 (14)	0.0024 (12)	0.0008 (13)
C75	0.0281 (19)	0.034 (2)	0.0297 (16)	−0.0002 (16)	0.0065 (13)	0.0040 (15)
C76	0.045 (2)	0.0214 (19)	0.0188 (14)	−0.0020 (16)	0.0003 (13)	−0.0021 (12)
C77	0.035 (2)	0.036 (2)	0.045 (2)	0.0035 (18)	0.0012 (16)	0.0009 (18)
N78	0.048 (2)	0.043 (2)	0.055 (2)	0.0035 (17)	−0.0035 (16)	0.0070 (17)
C79	0.037 (2)	0.046 (3)	0.0364 (18)	−0.0025 (18)	0.0058 (16)	0.0117 (17)
N80	0.049 (2)	0.059 (3)	0.053 (2)	0.0004 (18)	0.0093 (17)	0.0179 (18)
O81	0.0345 (14)	0.0291 (14)	0.0333 (12)	0.0036 (11)	−0.0001 (10)	0.0006 (10)
C82	0.040 (2)	0.031 (2)	0.0408 (19)	−0.0005 (17)	0.0075 (16)	0.0016 (16)
C83	0.048 (2)	0.041 (2)	0.0363 (18)	0.0029 (19)	0.0075 (17)	−0.0004 (16)
C84	0.057 (3)	0.042 (2)	0.045 (2)	0.007 (2)	0.0086 (18)	0.0043 (18)
O85	0.0384 (14)	0.0204 (12)	0.0249 (10)	−0.0030 (10)	0.0007 (9)	0.0034 (9)
C86	0.111 (4)	0.038 (3)	0.036 (2)	0.000 (3)	−0.004 (2)	0.0040 (18)
C87	0.073 (3)	0.038 (3)	0.050 (2)	0.005 (2)	0.003 (2)	0.0028 (19)
C88	0.059 (3)	0.047 (3)	0.053 (2)	0.000 (2)	0.001 (2)	0.016 (2)
O89	0.0347 (14)	0.0311 (14)	0.0322 (12)	−0.0015 (11)	0.0061 (10)	0.0018 (10)
C90	0.040 (2)	0.036 (2)	0.041 (2)	0.0012 (17)	0.0042 (16)	0.0030 (16)
C91	0.047 (2)	0.039 (2)	0.047 (2)	0.0018 (19)	0.0098 (18)	0.0048 (18)
C92	0.060 (3)	0.045 (3)	0.061 (3)	−0.002 (2)	0.002 (2)	0.013 (2)
O100	0.0472 (16)	0.0383 (16)	0.0376 (13)	0.0172 (13)	0.0110 (11)	0.0094 (11)
C101	0.049 (6)	0.025 (4)	0.039 (5)	−0.010 (4)	0.004 (3)	−0.005 (4)
C102	0.059 (4)	0.019 (4)	0.065 (5)	−0.001 (3)	0.026 (4)	−0.002 (3)
C103	0.053 (4)	0.039 (4)	0.078 (5)	−0.003 (3)	0.018 (4)	0.010 (4)
C201	0.050 (12)	0.039 (9)	0.047 (12)	−0.013 (8)	−0.013 (8)	−0.010 (9)
C202	0.050 (8)	0.029 (7)	0.028 (6)	0.009 (6)	−0.006 (6)	−0.012 (5)
C203	0.075 (12)	0.009 (8)	0.067 (10)	−0.015 (7)	−0.002 (8)	−0.002 (7)
O110	0.0448 (15)	0.0284 (14)	0.0214 (10)	0.0009 (11)	0.0019 (10)	0.0017 (9)
C111	0.034 (9)	0.054 (5)	0.022 (5)	−0.011 (8)	0.008 (6)	0.003 (4)
C112	0.098 (9)	0.031 (4)	0.032 (3)	0.005 (7)	0.009 (6)	0.003 (3)
C113	0.133 (11)	0.033 (6)	0.031 (5)	0.015 (6)	0.023 (4)	−0.004 (4)
C211	0.030 (12)	0.065 (7)	0.019 (6)	−0.004 (10)	0.010 (8)	0.012 (5)
C212	0.100 (12)	0.033 (6)	0.033 (5)	0.005 (10)	0.005 (9)	0.012 (4)
C213	0.130 (13)	0.037 (8)	0.045 (10)	0.018 (8)	0.000 (8)	0.003 (8)

### Geometric parameters (Å, °)

**Table:**

C1—C2	1.510 (4)	C57—C74	1.398 (5)
C1—C24	1.370 (5)	C58—C59	1.385 (5)
C1—C28	1.397 (5)	C58—H581	0.950
C2—C3	1.518 (5)	C59—C60	1.391 (5)
C2—H22	0.950	C59—H591	0.950
C2—H21	0.950	C60—C61	1.391 (5)
C3—C4	1.375 (5)	C60—H601	0.950
C3—C25	1.396 (5)	C61—C62	1.512 (5)
C4—C5	1.402 (5)	C61—C74	1.390 (5)
C4—H41	0.950	C62—C63	1.523 (4)
C5—C6	1.387 (5)	C62—H622	0.950
C5—C29	1.435 (5)	C62—H621	0.950
C6—C7	1.386 (5)	C63—C64	1.382 (5)
C6—H61	0.950	C63—C75	1.386 (5)
C7—C8	1.504 (5)	C64—C65	1.401 (4)
C7—C25	1.392 (5)	C64—H641	0.950
C8—C9	1.509 (5)	C65—C66	1.371 (5)
C8—H81	0.950	C65—C79	1.441 (6)
C8—H82	0.950	C66—C67	1.392 (5)
C9—C10	1.396 (5)	C66—H661	0.950
C9—C26	1.398 (5)	C67—C68	1.523 (5)
C10—C11	1.393 (6)	C67—C75	1.397 (4)
C10—H101	0.950	C68—C69	1.517 (5)
C11—C12	1.384 (6)	C68—H681	0.950
C11—H111	0.950	C68—H682	0.950
C12—C13	1.380 (5)	C69—C70	1.378 (5)
C12—H121	0.950	C69—C76	1.382 (5)
C13—C14	1.517 (5)	C70—C71	1.380 (6)
C13—C26	1.408 (5)	C70—H701	0.950
C14—C15	1.505 (5)	C71—C72	1.390 (6)
C14—H142	0.950	C71—H711	0.950
C14—H141	0.950	C72—H721	0.950
C15—C16	1.385 (5)	C73—O81	1.369 (4)
C15—C27	1.393 (5)	C74—O85	1.380 (4)
C16—C17	1.383 (5)	C75—O89	1.378 (4)
C16—H161	0.950	C76—O110	1.379 (4)
C17—C18	1.383 (5)	C77—N78	1.149 (5)
C17—C31	1.462 (6)	C79—N80	1.148 (5)
C18—C19	1.397 (5)	O81—C82	1.434 (4)
C18—H181	0.950	C82—C83	1.493 (5)
C19—C20	1.508 (5)	C82—H822	0.950
C19—C27	1.406 (5)	C82—H821	0.950
C20—C21	1.517 (5)	C83—C84	1.522 (6)
C20—H201	0.950	C83—H832	0.950
C20—H202	0.950	C83—H831	0.950
C21—C22	1.380 (5)	C84—H842	0.950
C21—C28	1.407 (4)	C84—H843	0.950
C22—C23	1.378 (5)	C84—H841	0.950
C22—H221	0.950	O85—C86	1.426 (5)
C23—C24	1.391 (5)	C86—C87	1.415 (6)
C23—H231	0.950	C86—H862	0.950
C24—H241	0.950	C86—H861	0.950
C25—O33	1.382 (4)	C87—C88	1.491 (6)
C26—O37	1.368 (4)	C87—H871	0.950
C27—O41	1.375 (4)	C87—H872	0.950
C28—O100	1.377 (4)	C88—H882	0.950
C29—N30	1.150 (5)	C88—H883	0.950
C31—N32	1.135 (5)	C88—H881	0.950
O33—C34	1.437 (4)	O89—C90	1.423 (4)
C34—C35	1.492 (5)	C90—C91	1.500 (5)
C34—H341	0.950	C90—H901	0.950
C34—H342	0.950	C90—H902	0.950
C35—C36	1.509 (6)	C91—C92	1.516 (6)
C35—H352	0.950	C91—H912	0.950
C35—H351	0.950	C91—H911	0.950
C36—H363	0.950	C92—H921	0.950
C36—H361	0.950	C92—H922	0.950
C36—H362	0.950	C92—H923	0.950
O37—C38	1.445 (4)	O100—C101	1.341 (8)
C38—C39	1.483 (6)	O100—C201	1.731 (19)
C38—H381	0.950	C101—C102	1.506 (11)
C38—H382	0.950	C101—H1011	0.950
C39—C40	1.510 (6)	C101—H1012	0.950
C39—H391	0.950	C102—C103	1.531 (9)
C39—H392	0.950	C102—H1022	0.950
C40—H403	0.950	C102—H1021	0.950
C40—H402	0.950	C103—H1031	0.950
C40—H401	0.950	C103—H1033	0.950
O41—C42	1.445 (4)	C103—H1032	0.950
C42—C43	1.494 (5)	C201—C202	1.48 (3)
C42—H421	0.950	C201—H2011	0.950
C42—H422	0.950	C201—H2012	0.950
C43—C44	1.522 (5)	C202—C203	1.53 (2)
C43—H432	0.950	C202—H2022	0.950
C43—H431	0.950	C202—H2021	0.950
C44—H443	0.950	C203—H2033	0.950
C44—H442	0.950	C203—H2032	0.950
C44—H441	0.950	C203—H2031	0.950
C49—C50	1.508 (5)	O110—C111	1.41 (2)
C49—C72	1.382 (5)	O110—C211	1.49 (3)
C49—C76	1.403 (5)	C111—C112	1.55 (3)
C50—C51	1.507 (5)	C111—H1111	0.950
C50—H501	0.950	C111—H1112	0.950
C50—H502	0.950	C112—C113	1.535 (15)
C51—C52	1.387 (5)	C112—H1122	0.950
C51—C73	1.403 (4)	C112—H1121	0.950
C52—C53	1.381 (5)	C113—H1133	0.950
C52—H521	0.950	C113—H1132	0.950
C53—C54	1.398 (5)	C113—H1131	0.950
C53—C77	1.442 (5)	C211—C212	1.45 (5)
C54—C55	1.383 (5)	C211—H2112	0.950
C54—H541	0.950	C211—H2111	0.950
C55—C56	1.506 (4)	C212—C213	1.41 (3)
C55—C73	1.402 (5)	C212—H2121	0.950
C56—C57	1.511 (5)	C212—H2122	0.950
C56—H562	0.950	C213—H2132	0.950
C56—H561	0.950	C213—H2131	0.950
C57—C58	1.389 (5)	C213—H2133	0.950
			
C2—C1—C24	120.5 (3)	C60—C59—H591	120.1
C2—C1—C28	121.4 (3)	C59—C60—C61	120.0 (3)
C24—C1—C28	118.1 (3)	C59—C60—H601	120.2
C1—C2—C3	114.9 (3)	C61—C60—H601	119.7
C1—C2—H22	107.0	C60—C61—C62	119.6 (3)
C3—C2—H22	107.5	C60—C61—C74	118.8 (3)
C1—C2—H21	109.1	C62—C61—C74	121.5 (3)
C3—C2—H21	108.8	C61—C62—C63	116.4 (3)
H22—C2—H21	109.5	C61—C62—H622	107.8
C2—C3—C4	120.4 (3)	C63—C62—H622	107.9
C2—C3—C25	121.0 (3)	C61—C62—H621	107.6
C4—C3—C25	118.6 (3)	C63—C62—H621	107.5
C3—C4—C5	120.4 (3)	H622—C62—H621	109.5
C3—C4—H41	119.9	C62—C63—C64	119.4 (3)
C5—C4—H41	119.7	C62—C63—C75	121.9 (3)
C4—C5—C6	119.7 (3)	C64—C63—C75	118.6 (3)
C4—C5—C29	120.0 (3)	C63—C64—C65	120.0 (3)
C6—C5—C29	120.3 (3)	C63—C64—H641	120.2
C5—C6—C7	121.0 (3)	C65—C64—H641	119.8
C5—C6—H61	119.4	C64—C65—C66	120.5 (3)
C7—C6—H61	119.6	C64—C65—C79	118.6 (3)
C6—C7—C8	120.5 (3)	C66—C65—C79	121.0 (3)
C6—C7—C25	118.0 (3)	C65—C66—C67	120.7 (3)
C8—C7—C25	121.5 (3)	C65—C66—H661	119.7
C7—C8—C9	117.1 (3)	C67—C66—H661	119.5
C7—C8—H81	107.3	C66—C67—C68	120.2 (3)
C9—C8—H81	107.3	C66—C67—C75	117.9 (3)
C7—C8—H82	107.9	C68—C67—C75	121.9 (3)
C9—C8—H82	107.7	C67—C68—C69	115.3 (3)
H81—C8—H82	109.5	C67—C68—H681	107.9
C8—C9—C10	119.6 (3)	C69—C68—H681	108.4
C8—C9—C26	122.2 (3)	C67—C68—H682	107.8
C10—C9—C26	118.2 (3)	C69—C68—H682	107.8
C9—C10—C11	121.4 (3)	H681—C68—H682	109.5
C9—C10—H101	119.0	C68—C69—C70	120.8 (4)
C11—C10—H101	119.5	C68—C69—C76	119.9 (3)
C10—C11—C12	119.2 (4)	C70—C69—C76	119.3 (4)
C10—C11—H111	120.3	C69—C70—C71	121.5 (4)
C12—C11—H111	120.4	C69—C70—H701	119.1
C11—C12—C13	121.1 (4)	C71—C70—H701	119.4
C11—C12—H121	119.6	C70—C71—C72	118.8 (4)
C13—C12—H121	119.3	C70—C71—H711	120.6
C12—C13—C14	121.3 (3)	C72—C71—H711	120.7
C12—C13—C26	119.2 (3)	C71—C72—C49	121.0 (4)
C14—C13—C26	119.5 (3)	C71—C72—H721	120.0
C13—C14—C15	114.8 (3)	C49—C72—H721	119.0
C13—C14—H142	108.0	C51—C73—C55	121.6 (3)
C15—C14—H142	108.0	C51—C73—O81	119.2 (3)
C13—C14—H141	108.4	C55—C73—O81	119.0 (3)
C15—C14—H141	108.1	C57—C74—C61	121.9 (3)
H142—C14—H141	109.5	C57—C74—O85	118.1 (3)
C14—C15—C16	120.1 (3)	C61—C74—O85	119.9 (3)
C14—C15—C27	122.3 (3)	C67—C75—C63	122.2 (3)
C16—C15—C27	117.6 (3)	C67—C75—O89	119.0 (3)
C15—C16—C17	121.3 (3)	C63—C75—O89	118.8 (3)
C15—C16—H161	119.6	C49—C76—C69	120.2 (3)
C17—C16—H161	119.1	C49—C76—O110	119.5 (3)
C16—C17—C18	120.5 (3)	C69—C76—O110	120.2 (3)
C16—C17—C31	119.6 (3)	C53—C77—N78	178.4 (4)
C18—C17—C31	119.9 (3)	C65—C79—N80	177.7 (4)
C17—C18—C19	120.2 (3)	C73—O81—C82	116.0 (3)
C17—C18—H181	119.4	O81—C82—C83	108.2 (3)
C19—C18—H181	120.4	O81—C82—H822	110.2
C18—C19—C20	120.0 (3)	C83—C82—H822	109.7
C18—C19—C27	117.9 (3)	O81—C82—H821	109.7
C20—C19—C27	122.1 (3)	C83—C82—H821	109.6
C19—C20—C21	115.8 (3)	H822—C82—H821	109.5
C19—C20—H201	107.5	C82—C83—C84	111.8 (3)
C21—C20—H201	108.0	C82—C83—H832	108.7
C19—C20—H202	108.0	C84—C83—H832	108.9
C21—C20—H202	108.0	C82—C83—H831	108.9
H201—C20—H202	109.5	C84—C83—H831	109.1
C20—C21—C22	120.7 (3)	H832—C83—H831	109.5
C20—C21—C28	121.2 (3)	C83—C84—H842	110.4
C22—C21—C28	118.1 (3)	C83—C84—H843	109.1
C21—C22—C23	122.1 (3)	H842—C84—H843	109.5
C21—C22—H221	118.7	C83—C84—H841	108.9
C23—C22—H221	119.2	H842—C84—H841	109.5
C22—C23—C24	118.2 (3)	H843—C84—H841	109.5
C22—C23—H231	120.7	C74—O85—C86	115.3 (2)
C24—C23—H231	121.1	O85—C86—C87	112.8 (4)
C23—C24—C1	122.4 (3)	O85—C86—H862	107.8
C23—C24—H241	119.0	C87—C86—H862	108.5
C1—C24—H241	118.6	O85—C86—H861	108.7
C3—C25—C7	122.1 (3)	C87—C86—H861	109.5
C3—C25—O33	118.8 (3)	H862—C86—H861	109.5
C7—C25—O33	119.1 (3)	C86—C87—C88	118.4 (4)
C13—C26—C9	120.7 (3)	C86—C87—H871	106.5
C13—C26—O37	118.6 (3)	C88—C87—H871	106.3
C9—C26—O37	120.7 (3)	C86—C87—H872	108.0
C19—C27—C15	122.3 (3)	C88—C87—H872	107.9
C19—C27—O41	117.0 (3)	H871—C87—H872	109.5
C15—C27—O41	120.6 (3)	C87—C88—H882	110.0
C21—C28—C1	121.1 (3)	C87—C88—H883	110.0
C21—C28—O100	118.5 (3)	H882—C88—H883	109.5
C1—C28—O100	120.4 (3)	C87—C88—H881	108.4
C5—C29—N30	179.1 (4)	H882—C88—H881	109.5
C17—C31—N32	177.7 (4)	H883—C88—H881	109.5
C25—O33—C34	113.6 (2)	C75—O89—C90	115.7 (3)
O33—C34—C35	109.9 (3)	O89—C90—C91	108.7 (3)
O33—C34—H341	109.6	O89—C90—H901	109.3
C35—C34—H341	109.6	C91—C90—H901	109.1
O33—C34—H342	109.7	O89—C90—H902	110.0
C35—C34—H342	108.6	C91—C90—H902	110.2
H341—C34—H342	109.5	H901—C90—H902	109.5
C34—C35—C36	111.2 (3)	C90—C91—C92	112.4 (3)
C34—C35—H352	108.4	C90—C91—H912	109.1
C36—C35—H352	108.2	C92—C91—H912	109.6
C34—C35—H351	109.6	C90—C91—H911	108.2
C36—C35—H351	109.9	C92—C91—H911	108.1
H352—C35—H351	109.5	H912—C91—H911	109.5
C35—C36—H363	110.7	C91—C92—H921	110.1
C35—C36—H361	109.7	C91—C92—H922	108.4
H363—C36—H361	109.5	H921—C92—H922	109.5
C35—C36—H362	108.0	C91—C92—H923	109.9
H363—C36—H362	109.5	H921—C92—H923	109.5
H361—C36—H362	109.5	H922—C92—H923	109.5
C26—O37—C38	114.9 (3)	C28—O100—C101	119.5 (4)
O37—C38—C39	108.7 (3)	C28—O100—C201	103.7 (8)
O37—C38—H381	109.3	O100—C101—C102	109.0 (6)
C39—C38—H381	109.4	O100—C101—H1011	109.2
O37—C38—H382	109.6	C102—C101—H1011	108.4
C39—C38—H382	110.3	O100—C101—H1012	111.7
H381—C38—H382	109.5	C102—C101—H1012	109.0
C38—C39—C40	114.7 (4)	H1011—C101—H1012	109.5
C38—C39—H391	108.2	C101—C102—C103	112.1 (6)
C40—C39—H391	107.9	C101—C102—H1022	107.8
C38—C39—H392	107.9	C103—C102—H1022	108.0
C40—C39—H392	108.5	C101—C102—H1021	108.3
H391—C39—H392	109.5	C103—C102—H1021	111.0
C39—C40—H403	109.8	H1022—C102—H1021	109.5
C39—C40—H402	109.7	C102—C103—H1031	111.8
H403—C40—H402	109.5	C102—C103—H1033	109.8
C39—C40—H401	109.0	H1031—C103—H1033	109.5
H403—C40—H401	109.5	C102—C103—H1032	106.8
H402—C40—H401	109.5	H1031—C103—H1032	109.5
C27—O41—C42	114.7 (2)	H1033—C103—H1032	109.5
O41—C42—C43	107.6 (3)	O100—C201—C202	105.8 (13)
O41—C42—H421	109.1	O100—C201—H2011	112.4
C43—C42—H421	109.3	C202—C201—H2011	115.4
O41—C42—H422	110.8	O100—C201—H2012	106.0
C43—C42—H422	110.6	C202—C201—H2012	107.2
H421—C42—H422	109.5	H2011—C201—H2012	109.5
C42—C43—C44	112.4 (3)	C201—C202—C203	114.7 (13)
C42—C43—H432	109.2	C201—C202—H2022	102.2
C44—C43—H432	109.2	C203—C202—H2022	112.2
C42—C43—H431	107.8	C201—C202—H2021	114.0
C44—C43—H431	108.6	C203—C202—H2021	104.5
H432—C43—H431	109.5	H2022—C202—H2021	109.5
C43—C44—H443	110.2	C202—C203—H2033	107.6
C43—C44—H442	108.8	C202—C203—H2032	106.4
H443—C44—H442	109.5	H2033—C203—H2032	109.5
C43—C44—H441	109.4	C202—C203—H2031	114.3
H443—C44—H441	109.5	H2033—C203—H2031	109.5
H442—C44—H441	109.5	H2032—C203—H2031	109.5
C50—C49—C72	120.7 (4)	C76—O110—C111	114.7 (10)
C50—C49—C76	120.4 (3)	C76—O110—C211	111.5 (16)
C72—C49—C76	118.9 (3)	O110—C111—C112	109.8 (15)
C49—C50—C51	118.4 (3)	O110—C111—H1111	110.6
C49—C50—H501	106.7	C112—C111—H1111	109.2
C51—C50—H501	106.7	O110—C111—H1112	110.4
C49—C50—H502	107.6	C112—C111—H1112	107.2
C51—C50—H502	107.8	H1111—C111—H1112	109.5
H501—C50—H502	109.5	C111—C112—C113	112.6 (11)
C50—C51—C52	119.6 (3)	C111—C112—H1122	107.9
C50—C51—C73	121.8 (3)	C113—C112—H1122	105.8
C52—C51—C73	118.6 (3)	C111—C112—H1121	111.8
C51—C52—C53	120.8 (3)	C113—C112—H1121	109.2
C51—C52—H521	120.2	H1122—C112—H1121	109.5
C53—C52—H521	119.0	C112—C113—H1133	111.1
C52—C53—C54	119.8 (3)	C112—C113—H1132	110.3
C52—C53—C77	119.4 (3)	H1133—C113—H1132	109.5
C54—C53—C77	120.8 (3)	C112—C113—H1131	107.0
C53—C54—C55	121.1 (3)	H1133—C113—H1131	109.5
C53—C54—H541	119.1	H1132—C113—H1131	109.5
C55—C54—H541	119.8	O110—C211—C212	107 (2)
C54—C55—C56	119.6 (3)	O110—C211—H2112	110.1
C54—C55—C73	118.0 (3)	C212—C211—H2112	116.6
C56—C55—C73	122.3 (3)	O110—C211—H2111	105.8
C55—C56—C57	116.3 (3)	C212—C211—H2111	107.7
C55—C56—H562	107.9	H2112—C211—H2111	109.5
C57—C56—H562	108.7	C211—C212—C213	113 (2)
C55—C56—H561	107.4	C211—C212—H2121	114.2
C57—C56—H561	107.1	C213—C212—H2121	112.5
H562—C56—H561	109.5	C211—C212—H2122	103.0
C56—C57—C58	121.5 (3)	C213—C212—H2122	103.9
C56—C57—C74	120.8 (3)	H2121—C212—H2122	109.5
C58—C57—C74	117.7 (3)	C212—C213—H2132	108.9
C57—C58—C59	121.2 (3)	C212—C213—H2131	105.3
C57—C58—H581	118.5	H2132—C213—H2131	109.5
C59—C58—H581	120.3	C212—C213—H2133	114.0
C58—C59—C60	120.1 (3)	H2132—C213—H2133	109.5
C58—C59—H591	119.9	H2131—C213—H2133	109.5

### Hydrogen-bond geometry (Å, °)

**Table:**

*D*—H···*A*	*D*—H	H···*A*	*D*···*A*	*D*—H···*A*
C10—H101···N30^i^	0.95	2.55	3.392 (8)	147

Symmetry codes: (i) *x*, *y*+1, *z*.
